# Intensive Foreign Language Learning Reveals Effects on Categorical Perception of Sibilant Voicing After Only 3 Weeks

**DOI:** 10.1177/2041669515613674

**Published:** 2015-12-09

**Authors:** Andreas Højlund Nielsen, Nynne Thorup Horn, Stine Derdau Sørensen, William B. McGregor, Mikkel Wallentin

**Affiliations:** Center of Functionally Integrative Neuroscience, Aarhus University Hospital, Denmark; Department of Linguistics, Cognitive Science and Semiotics & Interacting Minds Centre, Aarhus University, Denmark; Department of Linguistics, Cognitive Science and Semiotics, Aarhus University, Denmark; Center of Functionally Integrative Neuroscience, Aarhus University Hospital, Denmark; Department Linguistics, Cognitive Science and Semiotics & Interacting Minds Centre, Aarhus University, Denmark

**Keywords:** Foreign language learning, speech learning, perceptual adaptation, longitudinal

## Abstract

Models of speech learning suggest that adaptations to foreign language sound categories take place within 6 to 12 months of exposure to a foreign language. Results from laboratory language training show effects of very targeted training on nonnative speech contrasts within only 1 to 4 weeks of training. Results from immersion studies are inconclusive, but some suggest continued effects on nonnative speech perception after 6 to 8 years of experience. We investigated this apparent discrepancy in the timing of adaptation to foreign speech sounds in a longitudinal study of foreign language learning. We examined two groups of Danish language officer cadets learning either Arabic (Modern Standard Arabic and Egyptian Arabic) or Dari (Afghan Farsi) through intensive multifaceted language training. We conducted two experiments (identification and discrimination) with the cadets who were tested four times: at the start (T0), after 3 weeks (T1), 6 months (T2), and 19 months (T3). We used a phonemic Arabic contrast (pharyngeal vs. glottal frication) and a phonemic Dari contrast (sibilant voicing) as stimuli. We observed an effect of learning on the Dari learners’ identification of the Dari stimuli already after 3 weeks of language training, which was sustained, but not improved, after 6 and 19 months. The changes in the Dari learners’ identification functions were positively correlated with their grades after 6 months. We observed no other learning effects at the group level. We discuss the results in the light of predictions from speech learning models.

## Introduction

Models of foreign language speech learning predict that perceptual adaptation to foreign speech sound categories take place early in the process of acquiring a foreign language (Best, 1995; Best & Tyler, 2007; Flege, 1995). Best and Tyler (2007) suggest 6 to 12 months of experience as the primary window for perceptual adaptation to occur. Sound learning research using laboratory auditory training paradigms supports the notion of an early and rapid perceptual adaptation to foreign speech sound categories. However, they show effects of training on particular foreign language contrasts within just 1 to 4 weeks of training (e.g., Bradlow, Pisoni, Akahane-Yamada, & Tohkura, 1997; Chandrasekaran, Sampath, & Wong, 2010; Lively, Pisoni, Yamada, Tohkura, & Yamada, 1994; Logan, Lively, & Pisoni, 1991; Perrachione, Lee, Ha, & Wong, 2011; Wang, Spence, Jongman, & Sereno, 1999; Wong & Perrachione, 2007). This discrepancy in the timescales may simply be due to the different natures of the learning processes involved; general L2 learning encompasses new phonology, new vocabulary, potentially new syntax and even new pragmatics, whereas laboratory auditory training paradigms are directed at a few particular foreign phonetic contrasts and the learning of these contrasts are accomplished through very focused training. Thus, it seems that with focused exposure foreign language learners can achieve very rapid perceptual adaptation. For effects of linguistic immersion after 6 to 12 months of experience on perceptual adaptation, the evidence from behavioral cross-sectional studies is generally inconclusive (Best & Tyler, 2007; Moyer, 1999). There are, however, a few well-controlled cross-sectional studies that show effects of prolonged linguistic immersion when comparing groups of foreign language learners with different lengths of residence (LORs), namely ∼0.5 to 2 years and ∼6 to 8 years (Flege, Bohn, & Jang, 1997; Flege & Liu, 2001). The question thus remains: At what rate does perceptual adaption unfold in natural language learning settings? Does perceptual adaptation to the target language rapidly attune within the first couple of weeks with almost no further adaptation, or rather within the first 6 to 12 months of learning, and does adaptation increase further over an even longer timescale?

To answer this, we investigated perceptual adaptation in two groups of language officer cadets in a longitudinal study spanning 19 months of intensive language training. These cadets are a select group of highly motivated individuals who move from (almost) no knowledge to nearly complete fluency in a foreign language within the course of their 20-month-long language training. This provided us with a unique opportunity to study the full course of foreign language learning in a near-natural language learning setting within a realistic time frame.

### Time Course of Perceptual Adaptation

Two of the most central speech learning models, the perceptual assimilation model (PAM and PAM-L2; Best, 1995; Best & Tyler, 2007) and the speech learning model (SLM; Flege, 1995), both posit that perceptual adaptation to foreign speech sound categories occurs early in the process of acquiring a foreign language (Best & Tyler, 2007; Flege, 1995). Best and Tyler (2007) argue for their cutoff at 6 to 12 months of exposure by pointing to, on the one hand, Aoyama, Flege, Guion, Akahane-Yamada, and Yamada (2004) who found perceptual learning during 6 to 18 months rather than within the first 6 months of exposure; and on the other hand, the primacy of phonology as a building block and stepping stone for vocabulary and grammatical learning as a conceptual motivation for their perceptual adaptation window within 6 to 12 months of experience with the foreign language.

Flege et al. (1997) investigated effects of experience extending beyond the 6 to 12 months of exposure. They found more native-like perception of English vowels as an effect of prolonged LOR in the United States for four groups of nonnative English learners (native German, Spanish, Mandarin, and Korean speakers). The inexperienced groups’ mean LOR was 0.7 years, and the experienced groups’ mean LOR was 7.3 years. There are, however, several studies that do not show effects of prolonged experience with the foreign language on the learners’ speech perception and production (for reviews, see Best & Tyler, 2007; Moyer, 1999). In response to this, Flege and Liu (2001) conducted a study comparing two groups of Chinese students and two groups of Chinese nonstudents living in the United States on their perception of word-final English stops. This comparison revealed a beneficial effect of LOR on the perception of word-final English stops only between the two groups of students, not between the two groups of nonstudents, suggesting that the amount and quality of the exposure to the foreign language are crucial factors for perceptual adaptation.

Adaptation to foreign speech sounds has also been investigated in more experimental settings using targeted laboratory training paradigms varying in their degree of complexity of the tasks and stimuli used. Bradlow, Akahane-Yamada, Pisoni, and Tohkura (1999), Lively et al. (1994), and Logan et al. (1991) used relatively specific phonetic identification and discriminations tasks in combination with both speaker variability and non-synthesized (i.e., natural) stimuli for training Japanese learners on the English contrast between /r-l/. Chandrasekaran et al. (2010), Perrachione et al. (2011), and Wong and Perrachione (2007) used sound-to-meaning paradigms for teaching native English listeners to discriminate foreign lexical pitch contrasts (i.e., Mandarin pitch contours superimposed on English pseudowords). Common to both segmental contrast learning (Bradlow et al., 1999; Lively et al., 1994; Logan et al., 1991) and lexical pitch contrast learning (Chandrasekaran et al., 2010; Perrachione et al., 2011; Wang et al., 1999; Wong & Perrachione, 2007) was that the targeted laboratory training paradigms showed perceptual adaptation within just 1 to 4 weeks. Furthermore, the learning effects of these training regimes can be retained up to 3 and 6 months after training without sustained training (Bradlow et al., 1999; Lively et al., 1994; Wang et al., 1999).

To investigate the temporal discrepancy between the SLMs’ proposed window of 6 to 12 months of exposure for perceptual adaptation and the adaptation results from laboratory training paradigms within just 10 to 30 days, we tested the language officer cadets four times during their education: immediately before their language training started (T0), after 3 weeks (T1), after 6 months (T2), and after 19 months (T3). T0 was intended to reflect baseline with no or very minimal knowledge of the target language. T1 was intended to test the very early and rapid perceptual adaptation similar to the laboratory training paradigms. T2 was intended to test Best and Tyler’s (2007) early adaptation window of 6 to 12 months of experience. T3 was intended to examine the even later stages in the foreign language learning, nearly parallel to the later time intervals in Flege et al.’s (Flege et al., 1997; Flege & Liu, 2001) studies showing some effect of prolonged linguistic immersion.

To our knowledge, perceptual adaptation to the Arabic and Dari sound contrasts used in the present study has not been investigated in the context of foreign language sound learning thus far.

### Foreign Speech Sound Contrasts

The target languages for the two groups of participants were Arabic (Modern Standard Arabic [MSA] and Egyptian Arabic) for one group (*n* = 8) and Dari (Afghan Farsi) for the other (*n* = 12). For the choice of stimuli in the present study, we were, therefore, constrained by the Arabic phonology and the Dari phonology.

**Figure 1.**
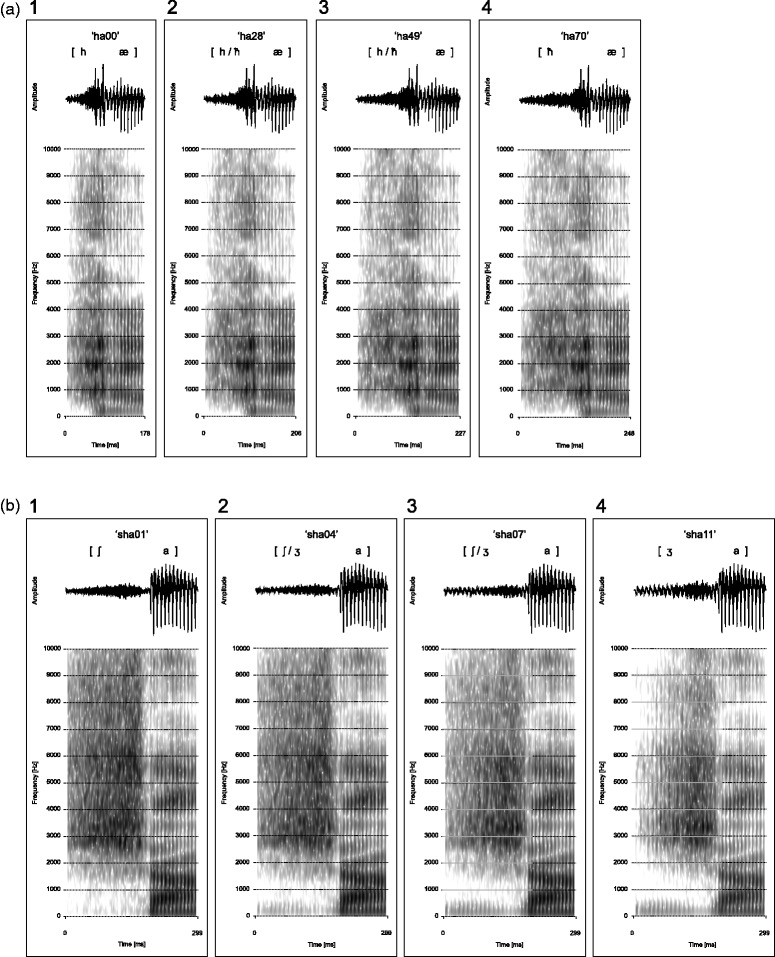
Spectrograms and amplitudes of both Arabic and Dari stimuli. (a) Arabic stimuli showing the four steps from the full 11-step continuum: (1) [hæ] + 0 ms of frication (“ha00”); (2) [hæ] + 28 ms of frication (“ha28”); (3) [hæ] + 49 ms of frication (“ha49”); and (4) [hæ] + 70 ms of frication (roughly equivalent to [ħæ], “ha70”). And (b) Dari stimuli also showing the four steps from the full 11-step continuum: (1) original [∫a] (“sha01”); (2) [∫/ʒa], more [∫] than [ʒ] (“sha04”); (3) [∫/ʒa], more [ʒ] than [∫] (“sha07”); and (4) original [ʒa] (“sha11”). Both sets of four sounds were used in the behavioral discrimination task. See Table 1 and the description of the stimuli in the text for fuller descriptions of the continua and the production of the stimuli.

We identified a phonemic sound contrast in each target language that was not phonemic in the other, or in Danish (the cadets’ mother tongue). This allowed us to treat the two groups as each other’s control group, which was a crucial feature of the experimental design since it would have been very difficult to find a suitable “active” control group. The Arabic stimuli consisted of the contrast between the glottal and pharyngeal fricatives (/h-ħ/), and the Dari stimuli consisted of the contrast between the voiced and voiceless sibilants (/ʒ-∫/; Figure 1).

We tested the cadets’ perceptual adaptation using two related experiments: an identification task (2AFC) and a discrimination task (same-different, i.e., AX). A steeper slope of the identification (ID) function indicates more consistent identification of stimuli into either of two (phonological) categories. Such categorical perception can be further substantiated by higher discrimination for stimulus pairs straddling the category boundary indicated by the ID function compared with pairs within either category.

On the basis of the models of speech learning and the research on laboratory auditory training, we hypothesized:

1. Identification: (a) that the slopes of the participants’ ID functions to the sound categories of their target language, but not their nontarget language, would become steeper as learning progressed. (b) That the steepening of the ID slopes would be detectable within the first 3 weeks of learning.
2. Discrimination: (a) that the participants’ discrimination peaks to the sound pairs of their target language would increase as learning progressed. This would not happen for any potential discrimination peak to the sound pairs of their nontarget language. (b) That the heightening of the discrimination peaks would be detectable within the first 3 weeks.

## Methods

The participants in the present study were tested both electrophysiologically (EEG) and behaviorally at each of the four times of measurement. The results of the EEG experiment will be reported elsewhere (Nielsen, Horn, Derdau Sørensen, McGregor, & Wallentin, in prep). The first measurement (T0) was in the very first week of the participants’ education. This first week was immediately before they started their language training (and right after 4 months of intensive physical training as sergeants). The three subsequent measurements were after 3 weeks (T1), 6 months (24 weeks; T2), and 19 months (81 weeks; T3). The fourth and final measurement (T3) was, thus, toward the very end of their 20-month-long intensive language training. The order of the two experiments was the same for all participants at all three measurements: the identification task first and the discrimination task last (and the behavioral tests were always after the EEG measurements).

The investigation was approved by the local ethics committee (De Videnskabsetiske Komitéer for Region Midtjylland), and written consent was obtained from each participant.

### Participants

Participants consisted of two groups of volunteer cadets from the Institute for Languages at the Royal Danish Defence College, enrolled in their first year of the language officer education at the onset of the study. One group was studying Dari (Afghan Farsi) and consisted of 12 participants (seven women, five men); the other group was studying Arabic (learning both MSA and Egyptian Arabic [Masri]) and consisted of nine participants of which one dropped out of the education and hence also the study after 4 weeks (*n* = 8; three women, five men). A total of 22 language officer cadets were enrolled in their first year when the study started. Of these 22, one dropped out (as already stated), and one (Arabic learner) did not participate in the study, otherwise all those enrolled participated. The two groups served as each other’s control groups since the stimuli were constructed in such a way that the Dari stimulus contrast was not phonemic in Arabic, and the Arabic stimulus contrast was not phonemic in Dari (see the Stimuli section below).

The participants had been prescreened by the Royal Danish Defence College on the basis of their school achievements, study skills, intelligence, and emotional stability among a large group of applicants (∼200) prior to enrollment into the 2-year program. All participants were native Danish listeners, and all had been hearing tested in relation to admission to the Royal Danish Defence College, on the basis of which they all reported having near-normal to normal hearing. None reported any history of neurological or psychiatric illness.

Throughout the 19 months, all 20 participants underwent comprehensive and intensive language training at the Institute for Languages at the Royal Danish Defence College. Intensive language learning in this respect entails language learning as a full-time job. The teaching was conducted both by native speakers and language experts of the relevant languages, and the curriculum involved many varied activities including extensive conversational practice with native speakers. The first 6 months focused almost exclusively on language learning, after which also teaching in cultural aspects and military strategy was included. After 3 weeks (T1), the cadets were familiar with the new sounds of their target language, and they had begun learning the writing system and acquiring vocabulary. After 6 months (T2), the cadets were proficient enough to conduct short conversations in their target language, and at the end of their education (T3) they were capable of functioning as interpreters and cultural experts in the field. We treated these levels of foreign language proficiency as basic (T1), intermediate (T2), and advanced (T3).

A few of the participants had some minor knowledge of either Arabic (*n* = 5) or Dari (*n* = 2) prior to enrollment (mainly in the form of 1- to 3-month-long stays abroad or evening classes during a semester; one Dari learner reported that his father was Iranian and spoke Farsi, but he clearly stated that he was not bilingual in relation to Farsi; and one Arabic learner had studied Arabic at the university for 3 years). At the initial measurement at T0, there were no significant differences between the group of learners with some prior knowledge of Arabic and the rest of the learners on either the slopes of their ID functions or the peaks of their discrimination functions in relation to the Arabic stimuli. Nor were there any significant differences between the two learners with some prior knowledge of Dari/Farsi and the rest of the learners at T0 on the same dependent variables in relation to the Dari stimuli (see the Results section for details on the statistics).

The two groups did not differ significantly on age (Dari, *M* = 24.2 [*SD* = 2.6]; Arabic, *M* = 23.4 [*SD* = 2.6]; *t*(18) = 0.64, *p* = .53), years of education (Dari, *M* = 16.7 [*SD* = 2.1]; Arabic, *M* = 16.0 [*SD* = 2.5]; *t*(18) = 0.67, *p* = .51), or number of foreign languages spoken (Dari, *M* = 3.6 [*SD* = 1.0]; Arabic, *M* = 4.1 [*SD* = 2.0]; *t*(18) = −0.80, *p* = .44). On the basis of Wallentin’s (2009) critical review of the putative gender differences in relation to language, we did not include gender as a matching criterion for the two groups.

### Stimuli

The stimuli varied in two contrasts, one which was phonemic in Dari and one which was phonemic in Arabic. The language officer cadets were expected to be excellent language learners (since they were prescreened on, among several other things, their language learning abilities). We, therefore, opted for contrasts that had been reported to be difficult for L2 learners to acquire.

The Dari contrast consisted of the sibilant voicing contrast between /∫/ and /ʒ/ (Entezar, 2010). For this choice, we relied on a combination of personal communication with the coordinating Dari teacher at the Institute for Languages (I. G. Smilianov, personal communication, July 23, 2012), and the phonologies of Danish (Basbøll, 2005; Grønnum, 2005), Dari (Entezar, 2010), and Arabic (Watson, 2007). The teacher reported that in his experience the cadets generally faced the most difficulties in acquiring the voicing contrast for the Dari sibilants (I. G. Smilianov, personal communication, July 23, 2012). Danish has a phonemic contrast between /s-ɕ/, but it has no voiced sibilants (Grønnum, 2005, 2009). [ʒ] exists as an allophone of the phoneme /ʤ/ in the Levantine dialects of Arabic (Watson, 2007), but the group of Arabic learners in the present study were learning MSA and Egyptian Arabic, in which the corresponding phoneme is realized as [ʤ] and [g], respectively (Watson, 2007). Hence the contrast’s lack of phonemic status for the Arabic learners was still upheld. We, therefore, opted for the /∫-ʒ/ contrast with the expectation that this would be the most difficult contrast in the Dari phonology for the Dari-learning cadets to successfully adapt to.

The Arabic contrast consisted of the contrast between /h/ and /ħ/ where /ħ/ is mainly realized with pharyngeal constriction and retracted tongue root (Bin-Muqbil, 2006; Laufer & Baer, 1988; Watson, 2007). The constriction of the lower pharynx results in a prolonged and strengthened frication for the pharyngeal fricative [ħ] compared with the glottal fricative [h] (Obrecht, 1968), as well as a lowering of the adjacent high vowels (/i/ and /u/), but only minimally for the low vowel /a/ used in the present study (Bin-Muqbil, 2006; Watson, 2007). For the choice of Arabic stimuli in the present study, we relied on a combination of a report by Burnham (2013) stating that the /h-ħ/ contrast is one of the most difficult, if not the most difficult contrast for learners of Arabic to acquire, and the phonologies of Danish (Basbøll, 2005; Grønnum, 2005), Dari (Entezar, 2010), and Arabic (Watson, 2007). Danish has a glottal fricative /h/, but no pharyngeal fricatives. The Dari alphabet includes the letters corresponding to both the Arabic pharyngeal fricative /ħ/ (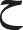) and the Arabic glottal fricative /h/ (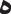), but neither of these are realized in spoken Dari (Entezar, 2010). On this basis, we opted for the pharyngeal-glottal fricative contrast with the expectation that this would be one of the most challenging contrasts for the Arabic-learning cadets.

Relevant Dari syllables ([∫a:] and [ʒa:]) were recorded from a male native Dari speaker using an SM58 Vocal Microphone (Shure Inc., Niles, IL, USA) in a sound-attenuated room. Relevant Arabic syllables ([hæl] and [ħæl]) were taken from recordings of a male native Arabic speaker.^1^ The Dari stimuli were then constructed by cross-splicing the recorded syllables so that the same vowel [a] with a duration of 109 ms was used for both Dari stimulus categories, resulting in [∫a] and [ʒa], both with a total duration of 299 ms, hence the duration of the sibilance was 190 ms (both voiceless and voiced). The same procedure was applied to the vowel in the Arabic stimuli so that the same vowel [æ] with a duration of 90 ms was used for both Arabic stimulus categories. The [h] was manipulated to resemble [ħ] by inserting 70 ms of frication from the [ħ]-recording into the [h] from 35 ms and onwards (see below for more details), resulting in [hæ] with a total duration of 178 ms and [hæ] + 70 ms with a total duration of 248 ms (roughly equivalent to [ħæ]). Since the participants were native Danish listeners and vowel length is phonemic in Danish (Basbøll, 2005, pp. 79–82; Grønnum, 2005, pp. 100–101), the duration of [æ] was not adjusted to compensate for the durational difference between [h] and [ħ].

Stimulus continua, each with 11 even-spaced steps, were created for both the Dari stimuli and the Arabic stimuli. For the Dari voicing contrast, we used the procedure described by Stevenson (1979) and Repp (1981) to mix the two sounds ([∫a] and [ʒa]) using 11 even-spaced levels of attenuation so that the first step from [∫a] to [ʒa] consisted of a mix between a completely unattenuated [∫a] and a completely attenuated [ʒa], thus rendering the original [∫a]. The next step from [∫a] to [ʒa] consisted of a mix between [∫a] attenuated by 0.92 dB and [ʒa] attenuated by 20.00 dB, corresponding to a 9/10- and a 1/10-power ratio of the two original sounds, respectively. This balance was step-wise reversed until the final step from [∫a] to [ʒa] consisted of a mix between a completely attenuated [∫a] and a completely unattenuated [ʒa], thus resulting in the original [ʒa] (see Table 1). This procedure for stimulus continuum generation has also been used by Broersma (2005, 2008, 2010) for voiceless-voiced fricative contrasts such as [s-z] and [f-v], acoustically very similar to the [∫-ʒ] used in the in present experiment. Also McQueen (1991), Norris, Mcqueen, and Cutler (2003), and Dufour, Kriegel, Alleesaib, and Nguyen (2014) have used the procedure for creating continua for voiceless fricative contrasts such as [f-s] and [s-∫].

**Table 1.** Gain Table for the Dari Stimuli, [∫a] and [ʒa].

	[∫a]										[ʒa]
Stimuli	sha01	sha02	sha03	sha04	sha05	sha06	sha07	sha08	sha09	sha10	sha11
[∫a] *L_W_* ratio	0	−1/10	−2/10	−3/10	−4/10	−5/10	−6/10	−7/10	−8/10	−9/10	−1
[ʒa] *L_W_* ratio	−1	−9/10	−8/10	−7/10	−6/10	−5/10	−4/10	−3/10	−2/10	−1/10	0
[∫a] dB gain	0.00	−0.92	−1.94	−3.10	−4.44	−6.02	−7.96	−10.46	−13.98	−20.00	−Inf
[ʒa] dB gain	−Inf	−20.00	−13.98	−10.46	−7.96	−6.02	−4.44	−3.10	−1.94	−0.92	0.00

*Note.* The continuum from [∫a] to [ʒa] was created by mixing the voiceless and voiced sibilant segments of the two original sounds (and thus endpoints of the continuum) with inversely proportional contributions from the two endpoints.

For the Arabic contrast, 70 ms of pharyngeal frication was taken from 24 to 94 ms into a realization of /ħæ/ and cut into 10 equally long bits of 7 ms. These cutouts were then one-by-one inserted into the frication of the glottal [hæ] from 35 ms into the sound and onwards, matching amplitude profiles of the frication and cutouts in order to avoid audible clippings in the sounds. This cross-splicing procedure resulted in an 11-step continuum ranging from [hæ] to a close approximation of [ħæ].

**Figure 2.**
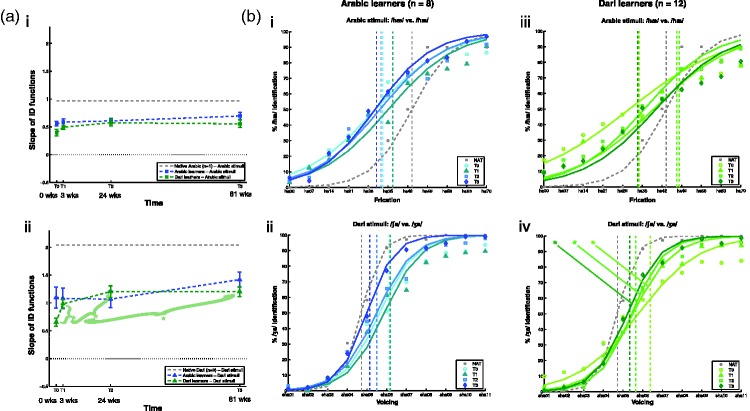
Participants’ identification functions to both sets of language stimuli across time. (a) Averages of the fitted slope values of participants’ identification functions to the Arabic stimuli (i) and Dari stimuli (ii) across the four times of measurement. Error bars show the standard error of the mean (*SEM*) with across participant differences removed (see Cousineau, 2005; Morey, 2008 for more details) in order to best reflect the tests that the significance asterisks refer to, namely the single-factor repeated measures ANOVAs. (b) Averaged observed values and identification functions interpolated from the averages of the participants’ slope and intercept values for each time of measurement and for each set of language stimuli. Darker colors denote later times of measurement. The vertical dashed lines mark the learners’ average category boundaries between the two stimulus categories. Data from one native Arabic listener for the Arabic stimuli (a, i) and (b, i–iii) and the averaged data from three native Dari listeners for the Dari stimuli (a, ii) and (b, ii–iv) are shown in gray as a reference for the learners’ responses. Asterisks denote significant effects of Time (*p* < .05).

**Figure 3.**
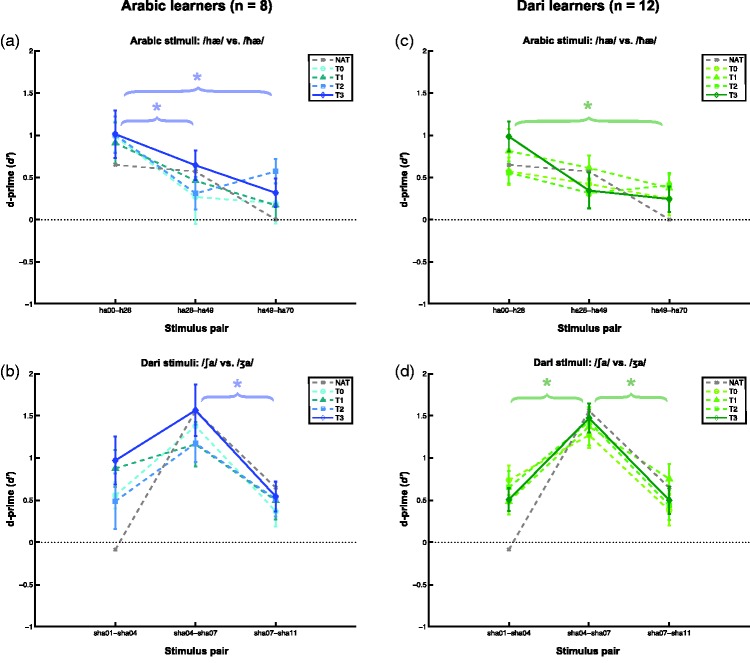
Mean discrimination data (*d*′) for the two groups across the four times of measurement and for each set of language stimuli. Darker colors denote later times of measurement. Asterisks denote significant main effects of Stimulus pair (*p* < .05). Data from one native Arabic listener for the Arabic stimuli (a) and (c) and the averaged data from three native Dari listeners for the Dari stimuli (b) and (d) are shown in gray as a reference for the learners’ responses. Error bars denote the standard error of the mean (*SEM*, no error bars are shown for the native listeners).

**Figure 5.**
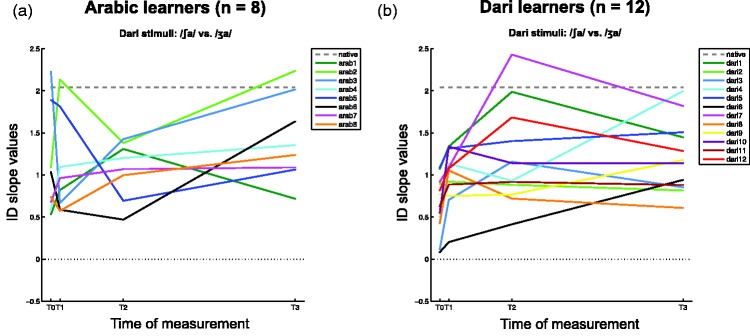
Individual identification slope values at all four times of measurement (T0–T3) for the Dari stimuli. The dashed gray lines represent the average of three native Dari listeners.

**Figure 6.**
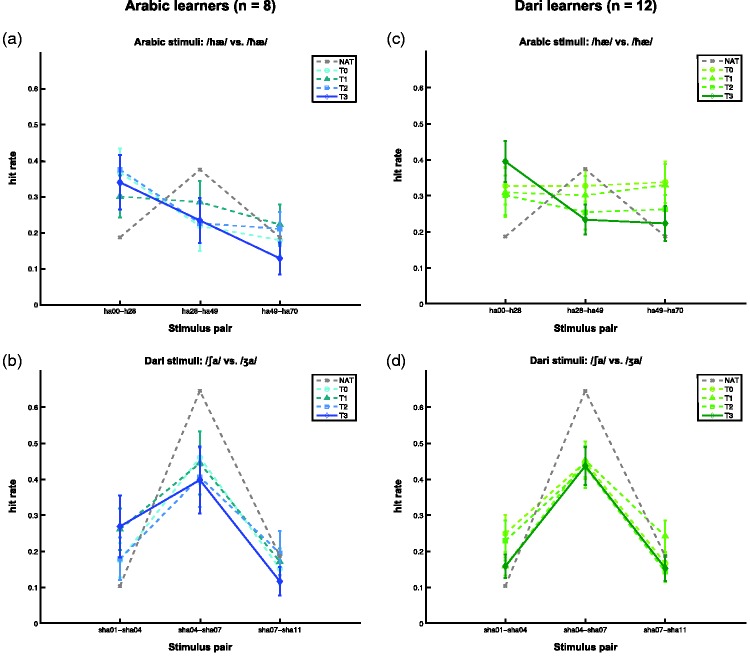
Mean hit rates for the two groups across times of measurement and for each set of language stimuli. Darker colors denote later times of measurement. Data from one native Arabic listener for the Arabic stimuli (a) and (c) and averaged data from three native Dari listeners for the Dari stimuli (b) and (d) are shown in gray as a reference for the learners’ responses. Error bars denote the standard error of the mean (*SEM*, no error bars are shown for the native listeners).

The stimuli were validated by one native Arabic listener and three native Dari listeners in a pilot study. They were presented with the same two experimental tasks as described below for the language learners. Instead of the arbitrary category labels of 1 and 2, they were presented with the written symbols corresponding to the relevant syllables in their native language (i.e., 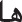 and 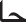 for Arabic and 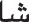 and 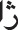 for Dari). The results of this pilot study are presented in gray dashed lines in Figures 2, 3, 5 and 6. The native Arabic listener showed a category boundary close to “ha42” which is evident from the gray vertical dashed lines in Figure 2(b-i, iii) and the peak in hit rates in Figure 6(a) and (c). The native Dari listeners showed an average category boundary close to “sha05” which is visualized by the gray vertical lines in Figure 2(b-ii, iv) and by the peak in *d*′ scores in Figure 3(b) and (d), as well as the peak in hit rates in Figure 6(b) and (d). The combination of steep ID functions and discrimination peaks straddling the category boundaries that both the native Arabic and Dari listeners showed for their respective stimuli validated the stimuli as representative exemplars from the phonologies of Arabic and Dari.

For the discrimination task, we selected two sounds from each of the stimulus continua together with the two endpoints (these were the same sounds that were used in the EEG experiment; Nielsen et al., in prep). For the Dari stimuli, these were thus “sha01”, “sha04”, “sha07”, “sha11”. The slightly asymmetric distribution was to ensure that the largest acoustic difference between either of the sound pairs did not straddle the category boundary identified by the native Dari listeners (i.e., “sha04-sha07” did not contain the largest acoustic difference). Otherwise, we could not have distinguished a potential effect of better discrimination across the category boundary from a potentially confounding effect of a larger acoustic difference. For the Arabic stimuli, the four selected sounds were “ha00”, “ha28”, “ha49”, “ha70”. Again, we ensured that the asymmetry in acoustic difference did not center on the category boundary identified by the native Arabic listener (i.e., “ha28-ha49” did not contain the largest acoustic difference).

### Task and Procedure

All participants were tested with two behavioral experiments: an identification task and a discrimination task. Each task had a Dari condition and an Arabic condition. The order of the two conditions within the tasks was counter-balanced across participants. We used Etymotic ER•2 insert earphones to deliver the auditory stimuli binaurally at a hearing level of approximately 80 dB. The execution and control of the stimuli presentation was carried out by Presentation software (Neurobehavioral Systems, Inc., Albany, CA, USA).

#### Identification

The identification task was a 2-alternative forced-choice task (2-AFC paradigm) and was preceded by two short exposures to each endpoint of the relevant stimulus continuum (Dari: “sha01” and “sha11”, presented as “1” and “2”, respectively; and Arabic: “ha00” and “ha70”, also presented as “1” and “2”, respectively). The use of arbitrary labels such as “1” and “2” instead of the corresponding orthographic symbols was to ensure that any potential effects of learning (i.e., time) were not confounded by the sheer effect of the participants learning the relevant alphabet (including the orthographic symbols for the sounds in question). In the identification task, the participants were presented with one randomly selected stimulus from the relevant 11-step continuum at a time, and they had been instructed to indicate by button presses on a keyboard whether the stimulus “sounded most like” either “1” or “2”. Each stimulus from the continuum was presented 12 times, resulting in a total of 132 trials distributed over three blocks.

#### Discrimination

The discrimination task consisted of a so-called *same-different task* (AX paradigm). Participants were presented with two randomly selected sounds from the relevant group of four sounds (preselected from each of the 11-step continua, see the Stimuli section). The task was to decide whether the two sounds sounded similar or different, again by button presses on a keyboard. The two sounds were presented with an interstimulus interval of 900 ms. Each unique pair of sounds was presented 8 times, resulting in 16 trials with each pair of different sounds (ignoring order) and 8 trials with each pair of completely identical sounds, resulting in a total of 128 trials distributed over four blocks. The stimulus pairs spanning more than three or four steps on the continuum (e.g., “ha00-ha49” or “sha04-sha11”) were only used as filler trials since they could not target any within category versus between category effects given that the native listener category boundaries were located within the “ha28-ha49”- and “sha04-sha07”-intervals. Participants were not informed of the objective frequencies of “same”/”different” trials (i.e., 32/96).

### Data Analysis

All data analyses of the behavioral data were conducted using MATLAB (R2010a, The MathWorks Inc., Natick, MA) and IBM SPSS Statistics version 20 for Mac OS X.

#### Identification

For each participant the slope and intercept of their ID functions were estimated by fitting a generalized logistic regression model to the individual participant’s responses with their identification response as the dependent variable (i.e., either “sound 1” or “sound 2”) and the stimulus as the independent variable (using the glmfit.m function in MATLAB, and using *logit* as the link function). This was done for both the Dari and the Arabic stimuli at all four times of measurement, thus leading to 2 × 4 sets of regression coefficients per participant. A shallower slope of the ID function indicates less sharp boundaries between the two phoneme categories, that is, a lower degree of consistency in assigning the different steps on the continuum to one of the two categories.

Before any further analyses, all participants’ ID functions were screened for label confusion. In case, the fitted slope to a participant’s responses was lower than −0.1 (i.e., a noticeably negative slope), and that the ID function by visual inspection also appeared “flipped”, we deemed that the given participant had answered as consistent as he or she could have, but had simply confused the labels, and we thus reversed the response scores so that all “1” button presses were made “2” button presses and vice versa. This was the case for five participants at T0 (three on the Dari stimuli and two on the Arabic stimuli), for four participants at T1 (two on the Dari stimuli and two on the Arabic stimuli), for two participants at T2 (one on the Dari stimuli and one on the Arabic stimuli), and for one participant at T3 (on the Dari stimuli).

#### Discrimination

For the analysis of the discrimination task only the participants’ responses to the identical pairs (e.g., “ha00-ha00” or “sha04-sha04”) and the “near” pairs (e.g., “ha00-ha28” or “sha07-sha11”) were included, that is, their responses to the filler pairs spanning more than three or four steps on the continuum (e.g., “ha00-ha49” or “sha04-sha11”) were not considered. For each participant at each of the four times of measurement we calculated their discrimination sensitivity as expressed by their d-prime (*d*′) scores (Macmillan & Creelman, 2005) to the three “near” pairs of both sets of stimuli. This was done for each stimulus pair by subtracting the *z*-value (normal deviate) of the mean proportional “same” responses to the relevant “same” pairs (e.g., “ha28-ha28” and “ha49-ha49”) from the *z*-value of the proportional “different” responses to the relevant “different” pairs (e.g., “ha28-ha49” and “ha49-ha28”). Since *z*-values are infinite for response rates of 0% and 100%, these were adjusted to 1/(2 × N) and 1−1/(2 × N), respectively, where N is the number of responses that the given response rate was calculated on (here N = 2 × 8 = 16 for both “near” pairs (e.g., “ha28-ha49” and “ha49-ha28”) and both “same” pairs (e.g., “ha28-ha28” and “ha49-ha49”; Macmillan & Creelman, 2005). One of the advantages of *d*′ scores over raw hit rates is that *d*′ scores take response bias into account and are thus proposed to better reflect the actual discrimination sensitivity of a given participant (e.g., Macmillan & Creelman, 2005). The larger the *d*′ score for a given “near” pair, the larger the discrimination sensitivity toward this particular “near” pair.

#### Statistical tests

Data were first analyzed using a mixed design analysis of variance (ANOVA) in SPSS with Group as between-subjects factor and Language and Time as within-subject factors for the identification task, and Language, Time, and Stimulus pair as within-subject factors for the discrimination task. We used post hoc comparisons to evaluate any significant effects from the mixed design ANOVA. Greenhouse-Geisser-correction of degrees-of-freedom values was used on the mixed design ANOVAs whenever Mauchly’s test of sphericity was significant. For the identification task, we used the participants’ slope values as our dependent variable. For the discrimination task, we used the participants’ *d*′ scores for each of the three stimulus pairs as our dependent variable.

Following from our hypotheses presented in the Introduction, the effects that we hypothesized would take place would be a three-way interaction of Group × Language × Time for the identification task, and a four-way interaction of Group × Language × Time × Stimulus pair for the discrimination task. Knowing that such complex interaction terms can prove difficult to interpret in full, we also a priori planned for four single-factor repeated measures ANOVAs for the identification data with Time as within-subject factor, one for each group of learners for each of the two sets of stimuli, in order to assess whether any learning effects would have taken place in either of the groups to either of the two sets of language stimuli. We, therefore, also a priori planned for four 2-factor repeated measures ANOVAs for the discrimination data with Time and Stimulus pair as within-subject factors, again one test for each group for each set of stimulus.

## Results

Both significant and nonsignificant main effects are reported, but for interactions and post hoc *t* tests only the relevant and significant effects are reported, as well as those explicitly stated in the hypotheses.

### Identification

There was a significant main effect of Language on the steepness of the slopes of the learners’ ID functions, *F*(1, 18) = 40.0, *p* < .001, with their slopes to the Dari stimuli being steeper than their slopes to the Arabic stimuli, suggesting that across the board both groups of learners more consistently partitioned the Dari stimulus continuum into two categories than they did with the Arabic stimulus continuum. There was also a significant main effect of Time on the learners’ slope values, *F*(3, 54) = 6.1, *p* = .001. Post hoc pairwise comparisons revealed that the learner’s slopes were overall steeper at T2 than at T0, *F*(1, 18) = 7.3, *r* = .54, *p* = .014, as well as at T3 compared with T0, *F*(1, 18) = 17.1, *r* = .70, *p* = .001. This means that on average across both groups and the two sets of stimuli, the learners’ showed steeper slopes after 6 and 19 months of learning, that is, improved categorical perception. However, the learning effects expressed in such a broad main effect of Time are difficult to disentangle from mere adaptation to the experimental paradigms. There was no significant main effect of Group, *F*(1, 18) = 1.3, *r* = .26, *p* = .27. There were no significant interaction effects. Keeping our initial hypotheses in mind, we explicitly note that the three-way Group × Language × Time interaction was thus not significant, *F*(2.5, 44.2) = 1.1, *r* = .16, *p* = .34. However, such complex three-way interaction terms are vulnerable to large individual differences within a limited number of participants as in the present study. We had, therefore, planned for four repeated measures ANOVAs to test for any learning effects within each group to either set of stimuli. These are presented in the following section.

#### Learning effects within each group

For any potential learning effects within each group to each set of stimuli not captured by the omnibus mixed design ANOVA, we only observed a significant learning effect within the group of Dari learners for the Dari stimuli. They showed a significant main effect of Time on the steepness of their slopes to the Dari stimulus continuum, *F*(3, 33) = 9.2, *p* < .001. Post hoc pairwise comparisons revealed that the Dari learners’ slopes were steeper at T1, *F*(1, 11) = 18.7, *r* = .79, *p* = .001, at T2, *F*(1, 11) = 17.5, *r* = .78, *p* = .002, and at T3, *F*(1, 11) = 21.1, *r* = .82, *p* = .001, compared with their slopes at T0; see Figure 2(a) and (b, iv). Hence, the Dari learners showed steeper slopes for their identification of the Dari stimuli already after 3 weeks of learning, and this effect was sustained after 6 and 19 months, but not further improved at the group level. We observed no significant effects of Time on the Arabic learners’ slopes for either the Arabic stimuli or the Dari stimuli, and no significant effect of Time on the Dari learners’ slopes for the Arabic stimuli.

In Figure 2(b), the fitted mean ID functions (“lines”) and the mean observed responses (“circles, triangles, squares, and diamonds”) for both groups to both sets of stimuli across all four measurements are visualized (darker colors reflect later times of measurement). The ID functions (lines) reflect group means of the slope and intercept values of the general logistic regression models that were first fitted to each participant’s responses. The circles, squares, triangles, and diamonds reflect the group averages of the participants’ responses to a given stimulus on either continuum (for a given time of measurement). The slopes (dashed lines) and observed responses (crosses) from the pilot data with native listeners of Arabic (*n* = 1) and Dari (mean of *n* = 3) used for stimulus validation are plotted in gray as a reference for the learners’ responses to the Arabic and Dari stimuli. We urge caution in interpreting the data from the native listeners since the number of participants is relatively low.

The dashed vertical lines reflect the averages of the stimulus values at the learners’ ID functions at 50% identification, more commonly referred to as their thresholds between the two more or less well-established categories. Note that since the fitted mean ID functions are interpolated from the group-averaged slopes and intercepts, the averaged thresholds do not necessarily intersect their corresponding fitted group ID functions at 50% identification. This is so because the averaged thresholds are based on averages of the individually estimated thresholds, whereas the corresponding fitted group thresholds would require estimation on the basis of the group-averaged slopes and intercepts. We opted for group averaging as close to the individual fits for all ID function parameters as possible, and therefore the visualizations carry an inherent discrepancy. Both groups’ thresholds are within the middle “near” pair for both sets of stimuli and at all times of measurement. For the Arabic stimuli, both groups’ average boundaries between stimulus categories are thus within “ha28-ha49”, and for the Dari stimuli within “sha04-sha07”.

In summary, we note that the effect of learning on the slopes of the Dari learners’ ID functions occurred already between T0 and T1, that is, within 3 weeks of starting the language training, and that this effect was still significant at T2 and at T3, that is, after 6 months and 19 months of language training, respectively. We further note that there were no further steepening of the Dari learners’ slopes from T1 and onwards.

### Discrimination

There was a significant main effect of Language on the learner’s *d*′ scores, *F*(1, 18) = 31.4, *p* < .001, with their overall *d*′ scores to the Dari stimuli being larger than those to the Arabic stimuli. There was also a significant main effect of Stimulus pair on the learners *d*′ scores, *F*(2, 36) = 12.5, *p* < .001. Post hoc pairwise comparisons revealed that the learners’ *d*′ scores were on average higher for the first pair (i.e., either “ha00-ha28” or “sha01-sha04”) than for the third pair (“ha49-ha70” or “sha07-sha11”), *F*(1, 18) = 9.6, *r* = .59, *p* = .006, as well as higher for the second pair (“ha28-ha49” or “sha04-sha07”) than for the third pair (“ha49-ha70” or “sha07-sha11”), *F*(1, 18) = 34.3, *r* = .81, *p* < .001. There was no significant main effect of Group, *F*(1, 18) < 1, *r* = .09, *p* = .71.

There was a significant interaction effect of Language × Stimulus pair, *F*(1.7, 30.7) = 27.1, *p* < .001. This indicates that the location of the peaks of the learners’ *d*′ scores differed between the two sets of stimuli. To explore this interaction effect, we conducted post hoc pairwise comparisons of each of the three combinations of stimulus pairs in one set of the stimuli to their corresponding combination of pairs in the other set of stimuli. These revealed two significant interactions: The learners’ *d*′ scores were higher for the second Dari pair (“sha04-sha07”) than for the first Dari pair (“sha01-sha04”) while their *d*′ scores were higher for the first Arabic pair (“ha00-ha28”) than for the second Arabic pair (“ha28-ha49”), *F*(1, 18) = 56.7, *r* = .87, *p* < .001. And the average difference between their *d*′ scores to the second and third pairs was larger for the Dari pairs than for the Arabic pairs, *F*(1, 18) = 33.4, *r* = .81, *p* < .001, however, both groups still showed higher *d*′ scores for the second than the third pair. There was also a marginally significant interaction between the first and third pairs between the two sets of stimuli, *F*(1, 18) = 4.3, *r* = .44, *p* = .053, trending toward a larger difference between *d*′ scores for the two pairs for the Arabic stimuli than for the Dari stimuli. These interaction effects suggest that the location of the learners’ discrimination peaks differed between the two sets of language stimuli. We examine the locations of these discrimination peaks in more detail in the following section.

There were no other significant interaction effects. And in relation to our initial hypotheses, we note that the four-way Group × Language × Time × Stimulus pair interaction was thus not significant, *F*(5.0, 90.1) = 1.4, *r* = .12, *p* = .227. Again, four-way interaction terms require a substantial number of participants in order to show significant effects if these effects are small as in the present study. We had therefore, as mentioned in the previous section, Statistical tests, planned for four repeated measures ANOVAs with Time and Stimulus pair as within-subject factors in order to test for any learning effects on the *d*′ scores within each group and each set of stimuli. These are presented in the following section.

#### Learning effects and discrimination peaks within each group

In our examination of the locations of the discrimination peaks within each group to each set of stimuli, we found significant main effects of Stimulus pair on their *d*′ scores for both groups to both sets of stimuli: Arabic learners on the Arabic stimuli (*F*(2, 14) = 6.4, *p* = .01); Arabic learners on the Dari stimuli, (*F*(2, 14) = 6.8, *p* = .008); Dari learners on the Arabic stimuli (*F*(2, 22) = 6.1, *p* = .008); and Dari learners on the Dari stimuli, (*F*(2, 22) = 16.3, *p* < .001). We did not find any significant main effects of Time on the groups’ *d*′ scores for either of the two sets of stimuli, nor any significant interactions between Time and Stimulus pair in any of the four analyses.

These four main effects of Stimulus pair are explored in further detail with post hoc pairwise comparisons in the following. The Arabic learners showed significantly higher *d*′ scores for the first Arabic pair (“ha00-ha28”) than for both the second Arabic pair (“ha28-ha49; *F*(1, 7) = 12.4, *r* = .80, *p* = .01) and the third Arabic pair (“ha49-ha70”; *F*(1, 7) = 6.5, *r* = .69, *p* = .04), regardless of Time. This suggests that the Arabic learners were more sensitive to the contrast in the first pair than to the contrasts in the second and third Arabic pairs. The location of their discrimination peak on the first Arabic pair was not in accordance with the averages of their category boundaries estimated on the basis of their ID functions’ slopes and intercepts (compare the peak in *d*′ scores located within the first Arabic pair in Figure 3(a) with the locations of the dashed vertical lines in Figure 2(b, i), located around “ha35”, i.e., within the second Arabic pair).

For the Dari stimuli, the Arabic learners’ *d*′ scores were significantly higher for the second Dari pair (“sha04-sha07”) than for the third Dari pair (“sha07-sha11”; *F*(1, 7) = 30.0, *r* = .90, *p* = .001). The Arabic learners thus showed half of a discrimination peak for the Dari stimuli. A full discrimination peak would have required that the Arabic learners’ *d*′ scores for the second Dari pair (“sha04-sha07”) were also significantly higher than for the first Dari pair (“sha01-sha04”), which was not the case (they only showed a weak trend toward significance), *F*(1, 7) = 4.5, *r* = .062, *p* = .072). Nonetheless, the Arabic learners’ highest *d*′ scores were for the second pair, and this location of their partial discrimination peak was in accordance with their average category boundaries based on their ID functions (in Figure 3(b), their peak in *d*′ scores are located at the second Dari pair, and in Figure 2(b, ii), the dashed vertical lines are located around “sha05” and “sha06”, i.e., also within the second Dari pair).

The Dari learners showed significantly higher *d*′ scores for the first Arabic pair (“ha00-ha28”) than for the third Arabic pair (“ha49-ha70”; *F*(1, 11) = 9.0, *r* = .67, *p* = .01), and marginally significantly higher than for the second Arabic pair (“ha28-ha49”; *F*(1, 11) = 4.7, *r* = .55, *p* = .054). This reflects an almost full discrimination peak located at the first Arabic pair (“ha00-ha28”), much as with the group of Arabic learners. Also for the Dari learners, this location of the peak was not in accordance with their average category boundaries based on their ID functions (in Figure 3(c), their peak in *d*′ scores are located at the first Arabic pair, whereas the dashed vertical lines in Figure 2(b, iii) are located around “ha35” and “ha49”, i.e., within the second, and not the first, Arabic pair).

For the Dari stimuli, the Dari learners showed significantly higher *d*′ scores for the second Dari pair (“sha04-sha07”) than for both the first Dari pair (“sha01-sha04”; *F*(1, 11) = 36.4, *r* = .79, *p* < .001), and the third Dari pair (“sha07-sha11”; *F*(1, 11) = 24.8, *r* = .73, *p* < .001). This reflected thus a full discrimination peak for the Dari learners at the second Dari pair (“sha04-sha07”), the location of which was in accordance with their average category boundaries based on their ID functions (in Figure 3(d), their peak in *d*′ scores are located at the second Dari pair, and the dashed vertical lines in Figure 2(b, iv) are located around “sha06”, i.e., also within the second Dari pair).

The above-mentioned discrimination peaks are illustrated in Figure 3 with the learners’ *d*′ scores for each set of stimulus pairs over the course of four measurements. The *d*′ scores from native listeners of Arabic (*n* = 1) and Dari (mean of *n* = 3) are plotted in gray as a reference for the Arabic in Figure (3(a) and (c)) and Dari (3(b) and (d)) stimuli, respectively. Again, we urge caution in interpreting the data from the native listeners since the number of participants is relatively low.

In short, we observed partial or full discrimination peaks for both groups to both sets of stimuli. These only overlapped with the group-averaged category boundaries for the Dari stimuli, suggesting strongest categorical perception for this contrast. We did not find any effects of learning in either of the four the analyses. In the first section of the Discussion, we briefly discuss this absence of learning effects in the discrimination task.

### Correlations Between Grades and Rates of Change

After both 17 weeks and 40 weeks of language training, the cadets were evaluated by an internal exam at the Institute for Languages. They were evaluated on four tasks: listening, speaking, reading, and writing. Since we investigated foreign language sound learning, we focused on the grades from the listening and the speaking task. The grades were given on the Danish grading scale ranging from −3 to 12,^2^ which we treated as an ordinal scale, rather than an interval scale, due to its somewhat peculiar distribution of grades (−3, 00, 02, 4, 7, 10, 12, where 02 is the lowest grade needed to pass an exam).

**Figure 4.**
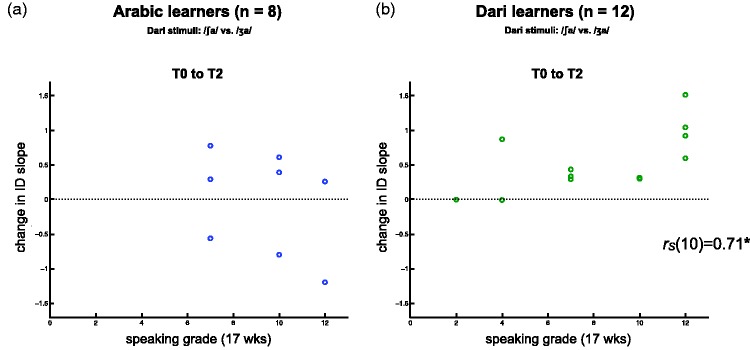
Correlations between changes in slope for the Dari stimuli from T0 to T2 and grades on a speaking task after 17 weeks (approx. at T2). (a) Arabic learners’ changes in the slopes of their identification functions for the Dari stimuli from T0 to T2 did not correlate with their exam grades on a speaking task after 17 weeks. (c) Dari learners’ changes in the slopes of their identification functions for the Dari stimuli from T0 to T2 correlated positively with their exam grades on a speaking task after 17 weeks. The asterisk denotes Bonferroni-adjusted alpha levels of *p* < .0125.

To investigate potential correlations between the cadets’ sound learning effects and their more general learning outcomes, we conducted Spearman’s rank correlations on the two groups’ changes in slope to the Dari stimuli between T0 and T1, between T0 and T2, and between T0 and T3 with their grades for the listening and the speaking task. To limit the number of correlations that we investigated to a sensible number, we focused exclusively on the measures of sound learning where we had observed significant learning effects, hence only the changes in slope to the Dari stimuli. We thus conducted four correlations for each dependent measure (i.e., we correlated each measure of slope change per group to two exam tasks after both 17 and 40 weeks), and we therefore used Bonferroni-adjusted alpha levels of .0125 per test. These 24 correlations in total revealed one significant correlation. The Dari learners’ changes in slope to the Dari stimuli from T0 to T2 were positively correlated with their speaking grades after both 17 weeks: (*r*_S_(10) = 0.71, *p* = .01; Figure 4(b)). For comparison, the Arabic learners’ changes in slope to the Dari stimuli for the same time interval (T0 to T2) are also plotted against their speaking grades; there was no significant correlation between the two measures (Figure 4(a)). We observed no other significant correlations for either of the two groups, or time intervals or grades (not depicted).

### No Differences due to Prior Experience

At the initial measurement at T0, the group of learners (*n* = 5) with some prior knowledge of Arabic did not differ significantly from the rest of the learners on the slopes of their ID functions to the Arabic phonemic contrast (with prior experience, *M* = 0.60, *SD* = 0.27; without prior experience, *M* = 0.42, *SD* = 0.27; *t*(6.8) = 1.30, *p* = .24), or on the peak differences of their discrimination functions to the Arabic contrast (with prior experience, *M* = 0.89, *SD* = 0.68; without prior experience, *M* = 0.66, *SD* = 0.46; *t*(5.3) = 0.69, *p* = .52). Nor did the two learners with some prior knowledge of Dari/Farsi differ significantly from the rest of the learners at T0 on the slopes of their ID functions to the Dari phonemic contrast (with prior experience, *M* = 0.50, *SD* = 0.59; without prior experience, *M* = 0.87, *SD* = 0.51; *t*(1,2) = −0.85, *p* = .54), or on the peak differences of their discrimination functions to the Dari contrast (with prior experience, *M* = 1.39, *SD* = 0.26; without prior experience, *M* = 0.90, *SD* = 0.50; *t*(1.9) = 2.21, *p* = .16).

## Discussion

In the present study, we investigated linguistic perceptual adaptation in a longitudinal study on two groups of language officer cadets learning either Arabic or Dari. We hypothesized that both groups would show increases in identification slopes and discrimination peaks as learning progressed, specific to their target language. We observed that the group of Dari learners’ identification slopes to the Dari stimuli steepened as an effect of learning, which partly confirmed the first of the two hypotheses. This was not the case for the Arabic learners to either set of the stimuli. We did not find support for our second hypothesis regarding the effects of learning on the discrimination peaks for either group. We did, however, see significant discrimination peaks straddling the category boundary for both groups in response to the Dari stimuli (however, only partially significant for the Arabic learners).

The main finding of this study is thus that the group of Dari learners’ developed significantly stronger categorical perception of the Dari sound contrast between [∫] and [ʒ] already after 3 weeks of intensive language training (i.e., from T0 to T1), and that this effect was still significant after 6 and 19 months of intensive language training (i.e., at T2 and T3). This supports the large body of research on laboratory auditory training also showing perceptual adaptation within 1 to 4 weeks of focused training. Since the Dari learners’ showed no further significant learning effects between T1 and T2, T1 and T3, and T2 and T3, this suggests that the learning effect we have observed is an index of an early perceptual adaptation that may plateau already after 3 weeks, after which it is sustained throughout the continued language learning, but without further improvements. Such an interpretation is, in fact, in line with results from a study on Japanese learners of Australian English in Australia (Bundgaard-Nielsen, Best, & Tyler, 2011). The Japanese learners showed relatively high levels of L2 phonological acquisition after less than 12 weeks in Australia, but no significant improvements on their perceptual adaptation over the following 4 to 6 months of L2 exposure. The results of the present data, however, only partly support such an interpretation because of its limited set of test items, which does not provide us with a comparable overview of the Arabic and Dari learners’ acquired L2 phonologies. Also, see Figure 5 and the related discussion for more details on the individual differences in the time course of the learning effects.

The Dari learners’ perceptual adaptation to their target language sounds in the identification task was the only significant change over time we observed in the present study. The discrimination task did, however, reveal that both groups of Arabic and Dari learners showed significant peaks in their discrimination sensitivities (*d*′ scores) for the Dari stimuli, straddling the category boundaries estimated by their average thresholds—Figure 2(b, ii–iv), vertical lines—which is indicative of some degree of categorical perception of the two phonological categories (/∫/ and /ʒ/) in both groups of learners already at T0. These indications of some level of phonological categories in both groups at the outset may reflect influence from second and third languages of most of language officer cadets, namely English (Giegerich, 1992) and French (Fougeron & Smith, 1993), respectively, which both have sibilant voicing contrasts. The fact that we did not observe any learning effects for the Dari learners on these discrimination peaks may suggest that the intervals for the Dari stimuli were not fine-grained enough to detect the improvements in the Dari learners’ categorical perception of the Dari contrast which we saw in their performances on the identification task.

Both groups of learners also showed discrimination peaks for the Arabic stimuli, but to the stimulus pair nearest the [hæ]-prototype, namely “ha00-ha28”. This peak did not straddle the category boundaries estimated by their average thresholds—see Figure 2(b, i–iii), vertical lines. The “ha00-ha28” pair did, however, straddle the largest acoustic difference of the three pairs (i.e., 28 ms as compared with 21 ms for the two other pairs). Hence, this discrepancy in discrimination sensitivity remains inconclusive insofar that the effect may be due to the larger acoustic difference.

### Individual Differences

We only observed statistically significant learning effects for the group of Dari learners. However, visually inspecting Figure 2(a, ii) it seems that the Dari learners’ initial performances were somewhat lower (as well as at T1 and T3) than those of the Arabic learners. None of these differences between the two groups were statistically significant. One possible explanation for these numerical differences comes from inspecting the individual learning progressions of the two groups of learners (Figure 5). The numerical differences between the two group averages thus seem to be mainly due to effects of comparing two groups with large individual differences.

In Figure 5(a), arab3 and arab5 were clearly driving the Arabic learners’ higher slope values at T0. But their high initial performances were not sustained across the following three measurements. At T1, arab2 and arab5 showed native-like performances. And at T3, arab2 and arab3 (and to some extent arab6) excelled. As already mentioned above, arab3 and arab5 had perhaps already at T0 perceptually successfully adapted to the foreign Dari contrast due to their L2 experience with English and French and thus contributed with very steep slopes to the Arabic learners’ group average at T0. However, if this were so, we would expect arab3 and arab5 to consistently show native-like performances across the subsequent measurements, which they did not. Instead, the Arabic learners’ performances changed in both directions over time, and it is difficult to identify a consistent pattern in their performances. And crucially in this context, the group of Arabic learners did not show any statistically significant learning effects on the Dari stimuli over the course of 19 months of learning Arabic, whereas the Dari learners did, in fact, show a consistent increase in slope steepness to the Dari stimuli as an effect of learning (this applies to 11 of the 12 Dari learners, Figure 5(b)).

In Figure 5(b), we see that there were, in fact, a few individuals who showed native-like performances at the later recording sessions (dari7 and dari1 at T2, and dari4 and dari7 at T3). But crucially, the Dari learners’ changes in slope over time seem to show a more consistent pattern than those of the Arabic learners: From T0 to T1, all except for 1 of the 12 Dari learners showed some increase in their slope values (i.e., all except dari9). Hence, the Dari learners showed large differences in the degree to which they improved their slope values (but the majority of them showed improvements), as well as in the offsets of their initial performances. The lack of further statistically significant learning effects (especially between T1 and T2) may therefore be due to these individual differences, as well as differences in the time courses of their improvements (some plateaued earlier than others).

The large individual differences, which we see in Figure 5, are also reflected in the relatively large error bars in Figure 2(a, ii). Note that the error bars have been adapted to best reflect the repeated measures ANOVAs, and hence the differences that they express relate to the progression between times of measurement for the two groups of learners (see Cousineau, 2005; Morey, 2008 for more details). Individual differences in learning foreign language speech contrasts have been attested in several studies (Chandrasekaran et al., 2010; Golestani & Zatorre, 2009; Perrachione et al., 2011; Wong & Perrachione, 2007) showing clear patterns of successful and less successful (sound) learners. The present study, however, was not designed to investigate individual differences within the two groups.

Nonetheless, we obtained the grades from the cadets’ exams after 17 weeks and after 40 weeks. And the Dari learners’ grades on a speaking task showed a significant positive correlation with their changes in identification slope from T0 to T2 (i.e., over the first 6 months), larger changes correlating with higher grades (Figure 4). This suggests that some of the individual differences reflected in the relatively large error bars in Figure 2(a, ii) do reflect meaningful variation in the Dari learners’ degree of perceptual adaptation, as well as the pace at which they adapt. We observe these individual differences despite the fact that the two groups of language officer cadets form a highly select population of skilled language learners. Finally, the correlation between their changes in slope and their speaking grades, but not their listening grades, may be suggestive of a strong link between perception (adaptation) and production, as has also been shown in the laboratory auditory learning settings (Bradlow et al., 1997, 1999; Wang, Jongman, & Sereno, 2003).

### Native Listeners of Arabic and Dari

As already mentioned in the Results section, we urge caution in interpreting the responses from the native listeners of Arabic (*n* = 1) and Dari (mean of *n* = 3) shown in gray in Figures 2, 3 and 6 due to the small number of informants. Nonetheless, the native listeners seem to show higher slope values for their native contrasts than the two group averages of the Arabic and Dari learners. At the same time, we observe a noteworthy similarity between the native listeners’ and the learners *d*′ scores in the discrimination task. Such response patterns suggest ceiling effects on this task, which is likely to be the case for the Dari stimuli where the stimulus intervals seem to have been too large to properly detect the learning effects which we observed in the identification task. Hence, the intervals were perhaps also too large to properly discern the performances of skilled L2 learners and native listeners. Interestingly, in the participants’ raw hit rates (see Figure 6), the differences between the native listeners and the learners show up more clearly. Whether this discrepancy in the patterns of the *d*′ scores and the raw hit rates between the native listeners and the learners could be due to response biases in the small native listener samples cannot be determined on the basis of the present data. But the raw hit rates do suggest differences in the performances of the native listeners and the learners on the discrimination task.

### Differences Between the Stimuli

We are somewhat surprised to see that the group of Arabic learners consistently showed stronger categorical perception for the Dari contrast than for the Arabic contrast. But as already stated in the Introduction, Burnham (2013) reported that the Arabic contrast between the glottal and pharyngeal fricatives is a very difficult contrast for learners of Arabic to acquire. We are furthermore surprised not to see any adaptation to this contrast during the course of 19 months of intensive Arabic teaching. The coarticulation cues in the neighboring /i/ and /u/ vowels around /ħ/ (Bin-Muqbil, 2006) lead us to speculate that the Arabic learners relied more on these secondary cues than on the prolonged and strengthened pharyngeal frication within the fricative itself. And since Danish vowels include relatively subtle vowel contrasts (Basbøll, 2005; Grønnum, 2005), it may be that the Arabic learners to some extent could have drawn on their L1 phonology for the general identification of /ħ/ via cues in the neighboring vowels. This speculation remains to be investigated in further depth using more varied stimuli. And finally, following Flege et al.’s (1997; Flege & Liu, 2001) findings of continued perceptual adaptation after several years of linguistic immersion, it may be that these cadets would indeed show some perceptual adaptation to such subtle contrasts after full linguistic immersion during stationing in Arabic-speaking regions.

### Implications for SLMs

In the present study, we showed perceptual adaptation to a Dari sound contrast during intensive multifaceted language learning within a time frame similar to those reported by targeted laboratory training regimes (Bradlow et al., 1999; Chandrasekaran et al., 2010; Lively et al., 1994; Logan et al., 1991; Perrachione et al., 2011; Wang et al., 2003; Wong & Perrachione, 2007), that is, within 3 weeks. This is much earlier than the early and rapid perceptual adaptation suggested by Flege’s SLM (1995) and Best and Tyler’s PAM-L2 (Best & Tyler, 2007), which Best and Tyler tentatively place within 6 to 12 months of exposure. Hence, when motivation to learn and amount and quality of input are high, it seems that the time frame for perceptual adaptation during second language learning may indeed mirror that of targeted laboratory training paradigms.

We did not see a similar effect of early and rapid perceptual adaptation with the Arabic learners on the Arabic contrast. In fact, we did not observe any perceptual adaptation at any time interval with the group of Arabic learners. However, for the reasons stated above regarding secondary perceptual cues, we speculate that this was not due to an overall lack of perceptual adaptation, but mainly due to the primary acoustic cue of strengthened and lengthened pharyngeal frication not being sufficiently salient to them given the rich vowel inventory in their native language (Danish). Hence, their lack of perceptual adaptation on the Arabic contrast does not necessarily contradict the SLMs’ predictions of early and rapid adaptation. However, it would be interesting to test the cadets’ categorical perception of the Arabic contrast after prolonged linguistic immersion during stationing in Arabic-speaking regions. Perhaps some foreign contrasts are so difficult to acquire that they require very extensive exposure, even for skilled and motivated L2 learners.

We did, however, also observe considerable individual variation in when the strongest adaptation took place for the Dari learners; (see Figure 5(b)). Half of the Dari learners (*n* = 6) showed the greatest steepening of their slope from T0 to T1, one fourth (*n* = 3) between T1 and T2, and the last fourth (*n* = 3) between T2 and T3. Hence, even though we mainly show evidence in support of a rapid and early window for perceptual adaptation in foreign language learning in the present study, we also show that within a rather homogenous group of skilled language learners, half of them show greatest perceptual adaptation after the initial 3 weeks. We find it plausible to argue that the significant initial adaptation we observe at the group level reflects a general rapid perceptual adaptation to the target language, even earlier than that suggested by Best and Tyler (2007). Further improvements at the later time intervals (nonsignificant at the group level) seem to be more prone to individual variation, which could be a tentative explanation for the lack of consistency in results from cross-sectional immersion studies reported by Moyer (1999).

## Conclusion

To conclude, we observed perceptual adaptation to a voiced-voiceless sibilant contrast for Dari learners already within 3 weeks of intensive language training. This effect persisted through the following 5 to 18 months of language training, but did not increase any further in this time window. This is supportive of the notion of a rapid and early window of perceptual adaptation during foreign language learning, potentially even earlier than posited by the leading SLMs. Furthermore, the degree to which the Dari learners’ slopes changed over time correlated positively with their exam grades from a speaking task (but not with their grades from a listening task), suggestive of a strong perception-production link.

## Notes

1 Taken with permission from the CD-ROM accompanying the teaching material al-Kitāb fi ta’allum al-‘Arabīyah (Brustad, Al-Batal, & Tūnisī, 1995).

2
http://fivu.dk/en/education-and-institutions/the-danish-education-system/grading-system
